# Radiological findings of abnormalities associated with congenital Zika virus infection: conclusions from World Radiology Day 2016

**DOI:** 10.26633/RPSP.2017.133

**Published:** 2017-12-05

**Authors:** Kayiba Peggy Medlen, Andrea Poretti, Thierry A.G.M Huisman, Jonel Di Muro, Priscilla Butler, Paula Woletz, Pablo Jiménez

**Affiliations:** 1 Department of Health Systems and Services/Unit of Medicines and Health Technologies Radiological Health Program, Pan American Health Organization Washington, DC. United States of America Department of Health Systems and Services/Unit of Medicines and Health Technologies, Radiological Health Program, Pan American Health Organization, Washington, DC, United States of America.; 2 Radiology and Radiological Science Johns Hopkins University BaltimoreMaryland United States Radiology and Radiological Science, Johns Hopkins University, Baltimore, Maryland, United States.; † Deceased 20 March 2017 Deceased 20 March 2017 Deceased 20 March 2017.; 3 Hospital Luis Razzetti Hospital Luis Razzetti BarcelonaAnzoategui Bolivarian Republic of Venezuela Hospital Luis Razzetti, Barcelona, Anzoategui, Bolivarian Republic of Venezuela.; 4 American College of Radiology American College of Radiology RestonVirginia United States American College of Radiology, Reston, Virginia, United States; 5 Howard Community College Diagnostic Medical Sonography ColumbiaMaryland United States Howard Community College, Diagnostic Medical Sonography, Columbia, Maryland, United States

**Keywords:** Zika virus, Zika virus infection, diagnostic imaging, microcephaly, neuroimaging, tomography, x-ray computed, magnetic resonance imaging, radiography, ultrasonography, Latin America, Caribbean Region, Americas, Virus Zika, infección por el virus Zika diagnóstico por imagen, microcephalia, neuroimagen, tomografía computarizada por rayos x, imagen por resonancia magnética, ultrasonografía, Latino América, Región del Caribe, Américas, Zika virus, infecção pelo Zika virus, diagnóstico por imagem, microcephalia, neuroimagem, tomografia computadorizada por raios x radiografia, imagem por ressonância magnética, radiografía, ultrassonografia, Latino América, Região do Caribe, América

## Abstract

In July 2015, Brazil reported an association between Zika virus infection and Guillain-Barre syndrome, and then in October 2015, between Zika and microcephaly. Most countries and territories in the Region of the Americas were later affected by the virus, creating a public health emergency.

Each year, the Pan American Health Organization (PAHO), Regional Office of the World Health Organization commemorates World Radiology Day (WRD), which highlights the role of radiology in public health. In 2016, PAHO devoted its WRD efforts to the Zika infection. Experts and partners presented and discussed the various radiological findings of Zika infection, the crucial role of obstetric ultrasound in the screening and monitoring of abnormalities associated with confirmed Zika virus infection, and the appropriateness of utilizing other neuroimaging technologies to study brain abnormalities in neonates and infants with prenatal Zika virus infection. The conclusions of WRD 2016 recommend that upon confirmation, prenatal ultrasound be used as the main tool to investigate and monitor suspected cases, with subsequent multidisciplinary postnatal assessments that include neuropediatric clinical studies and relevant neuroimaging. Additionally, radiology technicians should be adequately trained and a quality assurance program should be implemented to ensure timely, safe, and accurate diagnosis.

## RESUMEN

En julio del 2015, Brasil informó una asociación entre la infección por el virus del Zika y el síndrome de Guillain-Barré y luego, en octubre 2015, entre la infección por el virus del Zika y la microcefalia. Posteriormente, la mayoría de los países y territorios de la Región de las Américas se vio afectada por el virus, lo que dio lugar a una emergencia de salud pública. Todos los años, la Organización Panamericana de la Salud (OPS), la Oficina Regional de la Organización Mundial de la Salud, conmemora el Día Mundial de la Radiología, en el que se reconoce la importancia de la radiología en la salud pública. En el 2016, la OPS dedicó las actividades en el marco del Día Mundial de la Radiología a la infección por el virus del Zika. Diversos expertos y asociados presentaron los distintos signos radiográficos de la infección por el virus del Zika, y debatieron sobre esto, así como sobre el papel fundamental que desempeñan las ecografías obstétricas en el tamizaje y el seguimiento de las anomalías asociadas con la infección por el virus del Zika confirmada, y sobre la pertinencia de utilizar técnicas de neurodiagnóstico por imágenes para estudiar las anomalías cerebrales en recién nacidos y lactantes con infección por el virus del Zika prenatal. Las conclusiones del Día Mundial de la Radiología del 2016 recomiendan que, tras la confirmación de la infección, se utilicen las ecografías prenatales como herramienta principal para investigar y hacer un seguimiento de los casos presuntos, seguida por una evaluación posnatal multidisciplinaria en la que se incluyan estudios clínicos neuropediátricos y las técnicas de neurodiagnóstico por imágenes pertinentes. Además, se debe capacitar adecuadamente a los técnicos de radiología y se debe ejecutar un programa de garantía de la calidad para asegurar el diagnóstico oportuno, seguro y certero.

## RESUMO

Em julho de 2015, o Brasil informou existir associação entre a infecção por vírus zika e síndrome de Guillain-Barré e, em outubro do mesmo ano, entre o vírus zika e microcefalia. A maioria de países e territórios na Região das Américas foi subsequentemente afetada pelo vírus, constituindo emergência de saúde pública. Todos os anos, a Organização Pan-Americana da Saúde (OPAS), Escritório Regional da Organização Mundial da Saúde (OMS) comemora o Dia Internacional da Radiologia destacando o papel da radiologia em saúde pública. Em 2016, a OPAS dedicou este dia à infecção por vírus zika. Especialistas e parceiros apresentaram e debateram os diversos achados radiológicos na infecção por vírus zika, o papel fundamental do exame de ultrassom obstétrico na detecção precoce e monitoramento de anomalias associadas à infecção confirmada e quando convém usar outras técnicas de neuroimagem para estudar as anomalias cerebrais nos recém-nascidos e lactentes com infecção pré-natal. O painel do Dia Internacional da Radiologia 2016 recomenda que, com a confirmação da infecção, o ultrassom pré-natal seja o principal método usado para investigar e monitorar os casos suspeitos, com subsequente avaliação pósnatal multidisciplinar incluindo estudos clínicos neuropediátricos e exames de neuroimagem. Além disso, o pessoal técnico em radiologia deve ser adequadamente capacitado e implantado um programa de garantia da qualidade para assegurar o diagnóstico preciso, seguro e oportuno.

World Radiography Day is celebrated annually on November 8th to commemorate the discovery of x-radiation by Wilhelm Conrad Rontgen in 1895. For the Pan American Health Organization/World Health Organization (PAHO/WHO), World Radiology Day (WRD) is an opportunity to increase public awareness of the role that radiology plays in public health. On 9 November 2016, a panel of experts that included a neuroradiologist, a perinatologist, obstetrician-gynecologists, medical physicists, and a sonographer from the Region of the Americas gathered to discuss the crucial role of obstetric ultrasound in screening and monitoring confirmed Zika-related abnormalities. The group also covered the ap-propriateness of using other neuroimaging technologies, such as computed tomography (CT) and magnetic reso-nance imaging (MRI), to study brain abnormalities in neonates and infants with prenatal infection.

Zika virus (ZIKV) is an arbovirus of the Flaviviridae family that is usually transmitted by a mosquito. It was initially detected in a primate in 1947. As of 2015, a total of 48 countries and territories in the Americas have confirmed autochthonous, vector-borne transmission of Zika virus disease, and 20 of these have reported confirmed cases of congenital syndrome associated with Zika infection (1). The virus spreads to humans primarily through infected *Aedes* species mosquitoes; in pregnant women, the unborn child is at risk of adverse outcomes, including brain abnormalities, such as microcephaly, and disrupted brain development, intracranial calcifications, and eye abnormalities (2).

In October 2013, an outbreak of ZIKV was reported in French Polynesia, with some cases producing severe neurological complications (3). In November 2015, the Ministry of Health of Brazil attributed the increased number of neonatal microcephaly cases in the country’s northeastern area to congenital ZIKV infection. The rapid spread of the virus convinced WHO to announce ZIKV infection as a “Public Health Emergency of International Concern” in February 2016 (4).

This report presents (a) neurologic findings associated with Zika infection; (b) neuroimaging modalities used in diagnosing cranial and intracranial abnormalities associated with intrauterine ZIKV infection; (c) radiation exposure from medical imaging; and (d) neuroimaging in areas with limited resources.

## DISCUSSION AND RECOMMENDATIONS

### Neurologic findings associated with Zika infection

Familiarity with the neurologic and neuroimaging findings of congenital ZIKV infection is crucial to making an early diagnosis when evaluating the fetuses and newborns of residents and travelers to areas where Zika is endemic. The risks inherent in the abnormal development of the brain in fetuses of infected mothers were initially detected in Brazil with the description of microcephaly cases and other pre- and postnatal findings. According to PAHO, 22 countries have confirmed cases of Congenital Syndrome associated with Zika Virus Infection (CASaZVI); (5). In Venezuela, more than 200 000 suspected cases of Zika and 2 200 confirmed cases of Zika infection have been reported, and possible microcephaly cases have been documented (6).

The Zika virus is thought to disrupt the development of brain structures that would otherwise have developed normally. For example, neuronal and glial proliferation occurs before the 10^th^ week of gestation. If the disruption occurs during this time, microcephaly, intracranial parenchymal calcifications (mostly located at the level of the cortico-medullary junction), and ventriculomegaly may develop. Disruption during the period of neuronal migration may result in the development of lissencephaly and heterotopia, while later disruption may be associated with polymicrogyria and focal cortical dysplasia (7). Additional neurologic findings associated with congenital ZIKV infection include ventriculomegaly, enlarged extra-axial cerebrospinal fluid spaces, cerebellar and brainstem hypoplasia, and white matter signal changes (most likely due to dysmyelination and resulting in secondary thinning of the corpus callosum). Arthrogryposis, ocular abnormalities, and redundant scalp skin have also been noted in some cases (7, 8).

The present findings describe autochthonous cases of epidemiological suspicion that according to literature fit with CSaZVI, i.e., microcephaly, calcifications (mostly at the level of the cortico-medullary junction, but also , basal, ocular), ventriculomegaly, dysgenesis of corpus callosum, simplified gyral pattern, cerebellar hypoplasia, enlarged cisterna magna, and early arthrogryposis (9). Prenatal confirmation and ultrasound monitoring of suspected cases of CSaZVI are recommended, with subsequent multidisciplinary postnatal assessment that includes neuropediatric clinical studies, relevant neuroimaging (transfontanel ultrasonography, computed tomography [CT], and magnetic resonance imaging [MRI]), and evoked potentials, among others. For appropriate pre- and postnatal advice, the main guidelines for patient management and timely identification of CSaZVI cases include minimizing exposure of pregnant women and proper handling of vectors, along with timely investigation of suspected cases by epidemiology, clinical, and paraclinical services and the correct use of perinatal ultrasound (10).

### Diagnosis of intrauterine Zika virus infection

Several neuroimaging modalities are available to diagnose cranial and intracranial abnormalities associated with intrauterine ZIKV infection (11- 13). As shown in Table 1, prenatal ultrasound is the main tool for investigating abnormal findings in the development of the central nervous system (CNS) of the fetus (14). The International Society for Ultrasound in Obstetric and Gynecologic (London, United Kingdom) in its publication, *Interim Guidance on Ultrasound for Zika Infection in Pregnancy* (15), recommends:

- Accurate assessment of gestational age.
- Baseline ultrasound scan, including biometry and assessment of fetal anatomy.
- Measurement of the lateral ventricles and transcerebellar diameter.
- Assessment of intracranial anatomy.
  - Fetal head circumference (HC) of 2 standard deviations below the expected mean for gestational age, intracranial calcifications, irregularly-shaped ventricular margins, ventriculomegaly, increased periventricular echogenicity, intraventricular adhesions, calcifications, callosal or vermian dysgenesis, small transcerebellar diameter, enlarged cisterna magna and/or increased amount of cere-brospinal fluid around the brain.
  - Repeat every 4-6 weeks if local resources permit.

**TABLE 1.** Advantages and disadvantages of radiology technologies used to screen and monitor abnormalities associated with Zika virus infection, 2017

Technology	Advantages	Disadvantages
Computed tomography (CT)	CT may be widely available. The examination is rapid and usually does not require sedation.	CT has limited ability to distinguish between tissues with subtle differences in densities. CT utilizes ionizing radiation, which may have short-term and/or long-term side effects and may have an impact on the developing brain
Magnetic resonance imaging (MRI)	MRI does not use ionizing radiation. It produces high quality images, both structural and functional.	MRI is more expensive and may not be widely available. It requires a longer acquisition time and sedation may be required.
Diagnostic ultrasound (US)	There is no ionizing radiation and no sedation is required. It is relatively inexpensive and can be used to image the fetus and to obtain serial images.	Because ultrasound waves have limited ability to penetrate calvarial bone, imaging can only be done on fetuses, neonates, and young infants with open fontanels. Examination of newborns and infants must use the fontanels as acoustic windows to examine the brain, and is therefore limited when evaluating the presence of abnormalities in the brain periphery. Most important, ultrasound is highly operator-dependent.

***Source:*** Prepared by the authors from the study data.

Some fetal intracerebral findings, such as the presence of calcifications, periventricular or intraventricular margins, intraventricular echogenicities, and irregularly-shaped lateral ventricles may go undetected prior to 14 weeks of gestational age.

### Radiation exposure from medical imaging

X-rays, CT computed tomography, mammography, nuclear medicine, and radiation oncology procedures have significantly improved the health of people worldwide. These procedures are used to detect, diagnose, and treat diseases. However, as with any medical procedure, there are risks; consequently, these procedures must be used wisely and cautiously. Radiation exposure from medical imaging is the largest contributor to population exposure, greater than any other artificial source of radiation. In spite of this, the probability that radiation exposure from a diagnostic imaging procedure will cause cancer is very low.

It is important to employ two basic radiation protection concepts for all patients (16). The first is “justifica-tion.” This means performing an examination only when clinically indicated, using the correct technology to minimize inappropriate use, and following relevant national or international referral guidelines. One such set of guidelines is the American College of Radiology’s (Reston, Virginia, United States) *Appropriateness Criteria*,® which ranks procedure types in order of appropriateness, along with Relative Radiation Levels; additionally, its women’s topics section includes an Assessment of Fetal Well-Being (17). To avoid unnecessary radiation exposure to the patient, both the referring and interpreting physicians must have a thorough understanding of the exam type required and its appropriateness. It is crucial that medical professionals be aware of the relative radiation levels of the various imaging technologies.

The second concept is “optimization.” Optimization ensures that the correct equipment is used and techniques (and radiation exposure) are adjusted based on patient size or body habitus. This is important for pediatric imaging, especially for CT. Optimization also includes minimizing repeats, awareness of estimated patient dose, utilizing appropriate shielding, and ensuring operators are well trained and qualified to perform the imaging. Since the developing fetus is more sensitive to radiation exposure, special considerations should be given to pregnant patients, including whether to use ultrasound or MRI. The magnitude and types of risks to the fetus depend on the gestational age and the dose. Risks are generally low, but may include lethality, developmental abnormities, central nervous system effects, or cancer.

There are radiation risks throughout pregnancy that are related to pregnancy stage and dose. Radiation risks are greater during organogenesis and in the early fetal period; risks are somewhat less in the second trimester; and least in the third trimester. Malformations have a threshold of 100- 200 mGy or higher and are typically associated with central nervous system problems. During 8- 25 weeks post-conception, the central nervous system is particularly sensitive to radiation. Fetal doses in excess of 100 mGy can result in some reduction of intelligence quotient. Fetal doses in the range of 1 000 mGy can result in severe mental retardation and microcephaly, particularly during weeks 8- 15, and to a lesser extent, at weeks 16- 25. Radiation has been shown to increase the risk for leukemia and many types of cancer in adults and children. The relative cancer risk may be as high as 1.4 (40% increase over normal incidence) due to a fetal dose of 10 mGy. For an individual exposed in utero to 10 mGy, the absolute risk of cancer at 0- 15 years of age is about 1 excess cancer death per 1 700 (18, 19).

It is also important to remember that there are similar risks to this population, even when no radiation from medical imaging is involved. Prenatal doses from most properly-performed diagnostic procedures present no measurable increase in risk of prenatal death, malformation, or impairment of mental status over the background risks. In general, fetal doses of 100 mGy are not reached even with three pelvic CT scans or 20 conventional diagnostic X-ray examinations; however, with procedures using high-dose levels, fetal dose and potential risks should be estimated by qualified individuals (18-21).

### Neuroimaging in areas with limited resources

Due to the limited access to some high end technologies, low income countries may face challenges in the screening and monitoring of abnormalities associated with confirmed Zika infection. However, basic X-rays and ultrasound examinations alone can be used to diagnose most diseases and conditions in areas of Latin America and the Caribbean when access to MRI or CT is limited.

Although ultrasound is safe, low cost, and portable, it is operator reliant and requires extensive knowledge for acquiring and interpreting images. Operators must be properly trained and have access to continuing education. Furthermore, imaging units may be outdated in developing countries and a thorough knowledge of the physics behind the technology is essential to produce optimal images in an efficient, safe, and cost-effective manner. Available resources should be used in an efficacious manner; for example, if the ultrasound unit fails to automatically calculate gestational age measurements, growth charts should be available for reference purposes. Ultrasound operators should adhere to established examination protocols, utilize consistent scan planes, demonstrate appropriate landmarks, and if local protocols are not available, obtain measurements as stipulated or set forth by accrediting bodies, such as the American College of Radiology or the American Institute of Ultrasound in Medicine. Moreover, educational resources should be made available, including didactic and hands-on training to strengthen the knowledge and skills of both experienced and new radiology professionals. If possible, collaboration or alliances should be established to allow remote consultation with experts.

### Conclusion

Radiology plays an important role in the screening, diagnosis, and management of many diseases and conditions in Latin America and the Caribbean; however, it is often used poorly and ineffectively. Upon confirmation of Zika infection in a pregnant woman, prenatal ultrasound should be the main tool for investigating and monitoring the suspected ZIKV case, with subsequent multidisciplinary postnatal assessments, including neuropediatric clinical study and relevant neuroimaging. For this purpose, additional sonography training and telemedicine should be considered, especially in remote areas. Well-trained professionals should be operating the imaging devices, and interpreting the images. Moreover, these technologies should be utilized prudently and appropriately to prevent unnecessary radiation exposure, minimize cost, and provide accurate and timely diagnosis of Zika- associated abnormalities.

### Disclaimer

 Authors hold sole responsibility for the views expressed in the manuscript, which may not necessarily reflect the opinion or policy of the *RPSP/PAJPH* and/or PAHO.
